# Probing the mechanism for hydrogel-based stasis induction in human pluripotent stem cells: is the chemical functionality of the hydrogel important?†

**DOI:** 10.1039/c9sc04734d

**Published:** 2019-11-11

**Authors:** M. Sponchioni, C. T. O'Brien, C. Borchers, E. Wang, M. N. Rivolta, N. J. W. Penfold, I. Canton, S. P. Armes

**Affiliations:** Department of Chemistry, University of Sheffield Dainton Building Sheffield S3 7HF UK s.p.armes@sheffield.ac.uk; Department of Biomedical Science, University of Sheffield Western Bank Sheffield S10 2TN UK; Department of Biochemistry and Molecular Genetics, University of Louisville Louisville Kentucky 40202 USA

## Abstract

It is well-known that pluripotent human embryonic stem cells (hPSC) can differentiate into any cell type. Recently, we reported that hPSC colonies enter stasis when immersed in an extremely soft hydrogel comprising hydroxyl-functional block copolymer worms (I. Canton, N. J. Warren, A. Chahal, K. Amps, A. Wood, R. Weightman, E. Wang, H. Moore and S. P. Armes, *ACS Centr. Sci.*, 2016, **2**, 65–74). The gel modulus and chemical structure of this synthetic hydrogel are similar to that of natural mucins, which are implicated in the mechanism of diapause for mammalian embryos. Does stasis induction occur merely because of the very soft nature of such hydrogels or does chemical functionality also play a role? Herein, we address this key question by designing a new hydrogel of comparable softness in which the PGMA stabilizer chains are replaced with non-hydroxylated poly(ethylene glycol) [PEG]. Immunolabeling studies confirm that hPSC colonies immersed in such PEG-based hydrogels do not enter stasis but instead proliferate (and differentiate if no adhesion substrate is present). However, pluripotency is retained if an appropriate adhesion substrate is provided. Thus, the *chemical functionality* of the hydrogel clearly plays a decisive role in the stasis induction mechanism.

## Abstract 2

It is shown that hydroxyl functionality is required to induce stasis in human embryonic stem cell colonies immersed within wholly synthetic block copolymer worm gels with comparable storage moduli. Thus gel softness does not appear to be an essential parameter for stasis induction.

## Graphical abstract

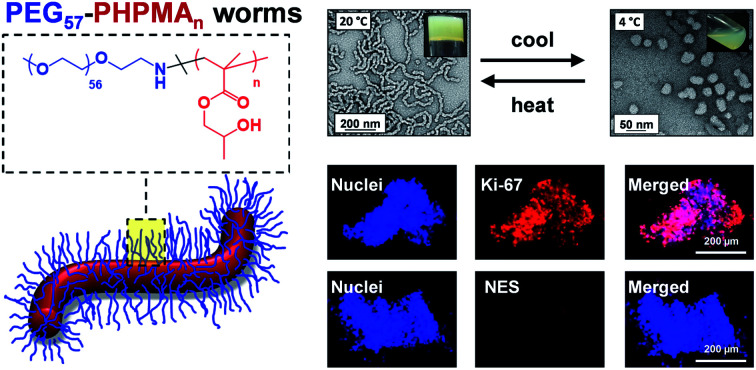

## Introduction

It is well-known that, given appropriate mechanical and (bio)chemical cues, pluripotent stem cells can proliferate^1^ and/or differentiate to form any desired cell type.^2,3^ Indeed, this is the basis of the field of regenerative medicine: if suitable 3D scaffolds are seeded with such cells and subjected to appropriate biochemical cues, then a wide range of tissues can be grown, including whole organoids.^4–6^ However, to retain pluripotency and prevent premature differentiation, continuous stem cell proliferation must be maintained. This is relatively straightforward within a well-controlled laboratory environment but becomes problematic during the global transportation of stem cells, which is essential unless clinical-grade stem cells can be cultured locally (*e.g.* within hospitals).^7^ In principle, this technical problem can be addressed by cryopreservation: stem cells can be frozen at 77 K using liquid nitrogen, transported to the point of use and thawed on demand. However, stem cells are relatively delicate: the majority of frozen cells either do not survive the thawing process or undergo differentiation after recovery: around 10–20% recovery of viable stem cells is typically achieved after cryopreservation.^8^ Moreover, shipment of cryogenically-frozen stem cells is relatively expensive *via* air freight. Considerable research effort has focused on the design of new synthetic 3D matrices for the culture of human stem cells. A range of synthetic vinyl copolymers with various functional groups^9–11^ as well as alginate^12^ and peptide-based^13^ hydrogels have been utilized as scaffolds for the continuous proliferation of stem cells. However, care must be taken to control gel stiffness because this parameter can affect stem cell differentiation.^14–16^ Interestingly, we reported that pluripotent embryonic human stem cells enter stasis (*i.e.* the G_0_ state of the cell cycle) if immersed as colonies within a new type of extremely soft wholly synthetic hydrogel.^17^ The majority of the quiescent stem cells survived in this environment for up to two weeks at 37 °C and expressed characteristic pluripotency markers (*e.g.* OCT4 and NANOG) on removal from the hydrogel. Importantly, this time period is sufficient to enable global transportation to any country *via* courier. This new synthetic hydrogel comprises highly anisotropic worm-like particles formed by the self-assembly of an amphiphilic poly(glycerol monomethacrylate)-poly(2-hydroxypropyl methacrylate) [PGMA-PHPMA] diblock copolymer.^18,19^ The worms are formed directly in aqueous solution during the synthesis of the diblock copolymer chains *via* a highly efficient and convenient process known as RAFT aqueous dispersion polymerization,^20–32^ which is an example of polymerization-induced self-assembly (PISA).^33–38^ Gelation is believed to be the result of multiple inter-worm contacts, which leads to local arrest of translational diffusion.^39^ Moreover, such hydrogels exhibit thermoresponsive behavior: they form low-viscosity, free-flowing fluids on cooling to 4 °C. This is because the worms are transformed into spherical micelles under these conditions.^40^ In principle, this thermoreversible morphology transition facilitates isolation of the stem cell colonies, as well as enabling convenient sterilization of the hydrogel *via* ultrafiltration of the cold fluid to remove bacteria.^40^

**Scheme 1:**
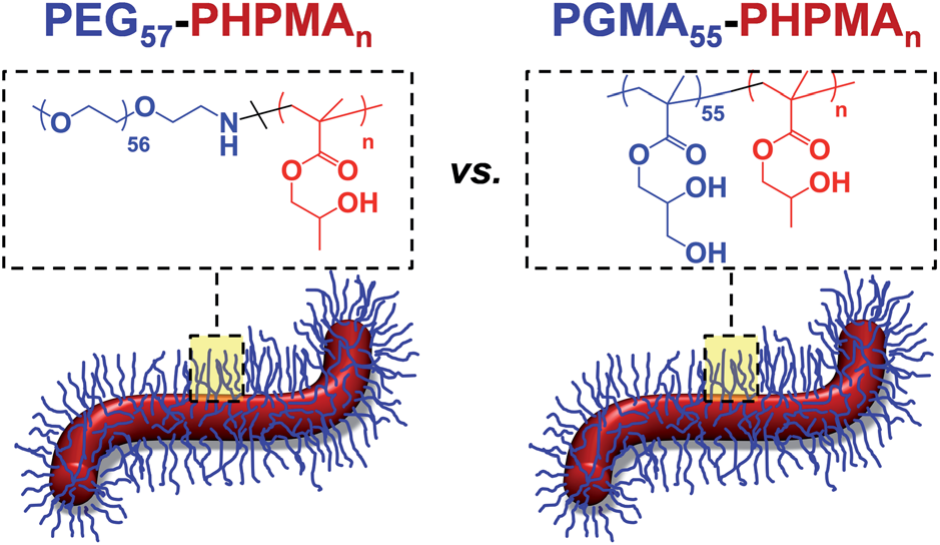
Schematic representation and chemical structure of (A) PEG_57_-PHPMA_120_ and (B) PGMA_55_-PHPMA_135_ diblock copolymer worms, which can form aqueous hydrogels of comparable mechanical strength at 37 °C.

It is currently not understood why pluripotent embryonic human stem cells enter stasis when placed within PGMA-PHPMA worm gels whereas tumoral cells^24^ and genetically abnormal human stem cells^17^ continue to proliferate under such conditions. Clearly, this is a fundamental scientific question that is likely to have broader implications. For example, an analogy can be drawn between such wholly synthetic hydrogels and naturally-occurring mucins, whereby secretion of the latter is believed to be important for the mechanism of delayed gestation (diapause) that is well-known for various mammalian embryos.^41^ There are two striking similarities between natural mucins and our wholly-synthetic worm gels: they both exhibit unusually low bulk gel moduli (*G*′ ∼ 10–50 Pa)^42^ and also possess hydroxyl functionality. Thus, in principle either mechanical and/or chemical cues could be responsible for inducing stasis in pluripotent human stem cells (hPSC). In the present study, we seek to differentiate between these two possibilities by designing a new type of synthetic hydrogel (see Scheme 1) whereby the many hydroxyl groups expressed at the surface of the worm-like nanoparticles are replaced with non-hydroxylated poly(ethylene glycol) (PEG) chains. PEG was selected because it has been widely used in many biomedical applications for several decades and is widely recognized to be highly biocompatible.^43–46^ The PEG-based worms have been designed to examine whether the hydroxyl-functional PGMA stabilizer block plays a critical role in inducing stasis in pluripotent human stem cell colonies. Thus, if PEG-based hydrogels also induce stasis in pluripotent embryonic human stem cells, this would suggest that the mechanical (rather than chemical) properties of the hydrogel are of paramount importance. On the other hand, if the stem cells continued to proliferate after their immersion in this new hydrogel this would provide strong evidence that the hydroxyl-rich nature of the PGMA stabilizer block (and, by implication, also that of natural mucins) plays an important, perhaps decisive, role in the stasis induction mechanism.

## Results and discussion

### Synthesis and characterization of PEG_57_-PHPMA_*n*_ diblock copolymer nanoparticles

**Fig. 1:**
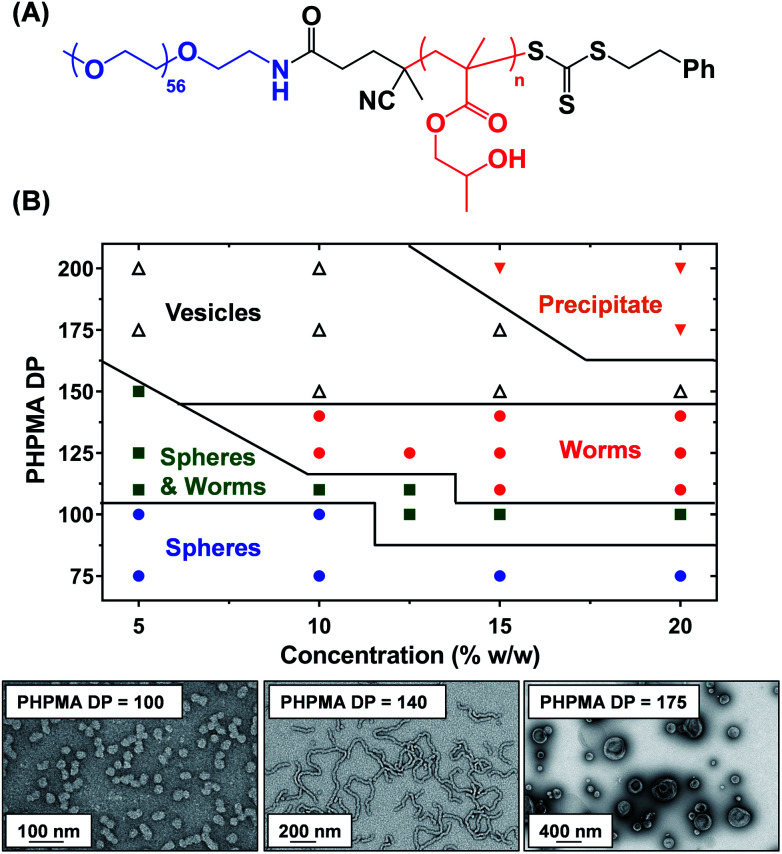
(A) Generic chemical structure for a series of PEG_57_-PHPMA_*n*_ diblock copolymers where *n* represents the target PHPMA DP. (B) Phase diagram constructed for such PEG_57_-PHPMA_*n*_ copolymers prepared *via* RAFT aqueous dispersion polymerization of HPMA at 40 °C. Representative TEM images obtained for PEG_57_-PHPMA_*n*_ spheres (*n* = 100), worms (*n* = 140) and vesicles (*n* = 175) prepared at 10% w/w copolymer concentration in each case.

**Fig. 2:**
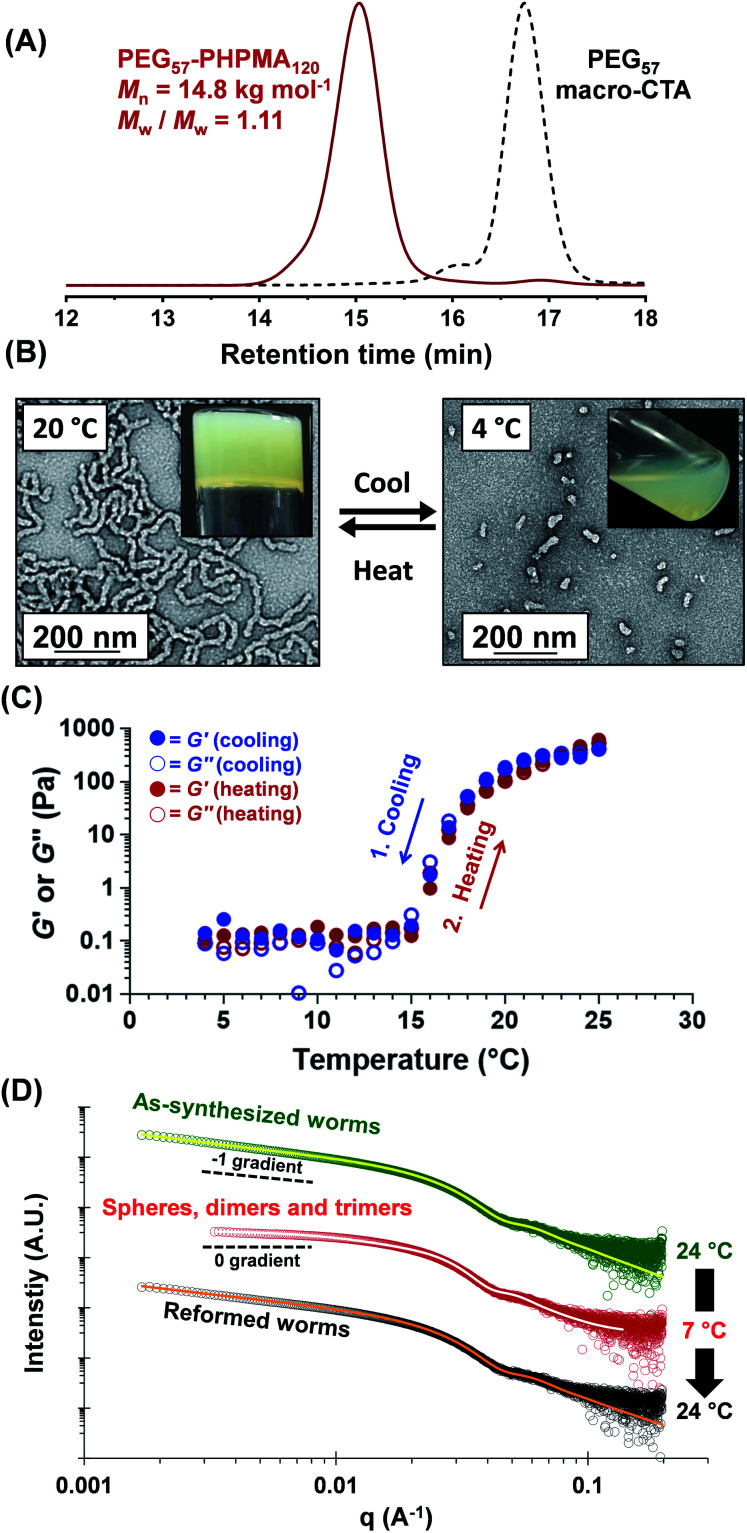
(A) DMF GPC curves obtained for a PEG_57_-PHPMA_120_ diblock copolymer and its corresponding PEG_57_ macro-CTA (molecular weight data are expressed relative to PEG standards). (B) TEM images and digital photographs for the thermoreversible degelation behavior exhibited by a PEG_57_-PHPMA_120_ worm gel, which undergoes a worm-to-sphere transition to form a free-flowing liquid when cooled to 4 °C. (C) Storage (*G*′) and loss moduli (*G*′′) obtained by oscillatory rheology measurements during a thermal cycle for a 10% w/w aqueous dispersion of PEG_57_-PHPMA_120_ nano-objects. This thermal cycle involved cooling from 25 °C to 4 °C (at 1 °C intervals) and then returning to 25 °C; data were obtained at an angular frequency of 1.0 rad s^−1^ at 1.0% strain, with 5 min being allowed for thermal equilibration between each measurement. (D) Representative *I*(*q*) *vs. q* plots recorded for 1.0% w/w aqueous dispersions of as-synthesized PEG_57_-PHPMA_120_ worms (green circles) at 24 °C, spheres, dimers and trimers (red circles) at 7 °C and reconstituted worms (black circles) at 24 °C. The data fits to the first and last SAXS patterns obtained using a worm-like micelle model are shown in yellow and orange, while the data fit obtained for the middle SAXS pattern utilized a ‘spheres, dimers and trimers’ model. In each case, the low *q* gradients (approximately −1 for worms and close to zero for spheres, dimers and trimers) are consistent with the corresponding TEM images.

A three-step amidation^47^ synthetic protocol, previously reported for a PEG_113_ macromolecular chain transfer agent (macro-CTA), was used to prepare a PEG_57_ macro-CTA (Fig. S1A†).^25,48^^1^H NMR spectroscopy studies indicated a mean degree of amidation of 93% (the integrated aromatic signals at 7.2–7.4 ppm were compared to that of the PEG_57_ backbone at 3.3–3.9 ppm, Fig. S1B†). Gel permeation chromatography (GPC) analysis (*N*,*N*′-dimethylformamide (DMF) eluent, PEG calibration standards, refractive index and UV detectors) indicated a number-average molecular weight (*M*_n_) of 2.4 kg mol^−1^ and an *M*_w_/*M*_n_ of 1.10 (Fig. S2†). This PEG_57_ macro-CTA was then chain-extended *via* RAFT aqueous dispersion polymerization of HPMA at 40 °C to yield PEG_57_-PHPMA_*n*_ diblock copolymer nanoparticles at 5–20% w/w solids, where *n* was varied from 75 to 200. Each diblock copolymer was analyzed by ^1^H NMR spectroscopy, DMF GPC and transmission electron microscopy (TEM) to assess the monomer conversion, molecular weight distribution and copolymer morphology, respectively (Table S1†). ^1^H NMR spectroscopy studies indicated more than 99% HPMA conversion in all cases. DMF GPC analysis indicated high blocking efficiencies for the original PEG_57_ macro-CTA (Fig. S2†) and unimodal molecular weight distributions, with *M*_w_/*M*_n_ values below 1.17 in most cases. As expected, targeting higher degrees of polymerization (DPs) for the PHPMA block resulted in a systematic increase in *M*_n_. TEM studies were performed using 0.1% w/w aqueous dispersions and a phase diagram (Fig. 1B) was constructed to examine how systematic variation of the copolymer concentration and target diblock composition affected the final copolymer morphology. Pure phases of worms and vesicles could be reproducibly obtained when targeting higher PHPMA DPs. Representative TEM images for spheres, worms and vesicles are displayed in Fig. 1B. PISA syntheses performed at higher copolymer concentrations (*i.e.* 15% w/w or 20% w/w) produced a broader worm phase. However, macroscopic precipitation was observed when targeting higher PHPMA DPs. This phase diagram is broadly consistent with literature data reported for PEG-based block copolymer nano-objects prepared *via* PISA.^25,27,49^ Importantly, construction of this phase diagram enables the reproducible synthesis of PEG_57_-PHPMA_*n*_ worm gels. Such worm gels must display degelation on cooling to allow stem cell stasis studies. Furthermore, it is *essential* that this transition is fully reversible so that the stem cell colonies can be easily recovered from the hydrogel. In 2014, the thermoresponsive behavior of similar PEG_113_-PHPMA_220_ worm gels was reported.^25^ As expected, degelation occurred on cooling from ambient temperature to 4 °C as a result of the concomitant worm-to-sphere transition.^39^ Unfortunately, this morphological transition proved to be *irreversible* when returning to room temperature, so regelation did not occur. This is in marked contrast to the well-documented thermoreversible behavior exhibited by PGMA-PHPMA worm gels.^19^ The relatively long PEG_113_ block confers steric stabilization during PISA, but does not prevent the 1D fusion of multiple monomer-swollen spheres that is required to form worms.^50^ However, in the *absence* of any unreacted HPMA monomer (which acts as an important processing aid during PISA),^51^ the steric stabilization bestowed by the PEG_113_ chains is sufficiently strong to prevent sphere–sphere fusion occurring, at least on normal experimental time scales (hours or days). In principle, lowering the mean DP of the PEG macro-CTA should modulate the steric repulsive forces between the cold spheres and hence enable a reversible worm-to-sphere transition to be achieved. We recently reported that using a binary mixture of a relatively long PEG_113_ and short PEG_45_ macro-CTAs provided access to thermoreversible worms.^49^ To examine this hypothesis, a large batch of PEG_57_-PHPMA_120_ worms was synthesized at 10% w/w solids. An HPMA conversion of more than 99% was confirmed by ^1^H NMR spectroscopy studies while DMF GPC analysis indicated an *M*_n_ of 14.8 kg mol^−1^ and an *M*_w_/*M*_n_ of 1.11 (Fig. 2A). According to the phase diagram shown in Fig. 1, this diblock composition should afford a pure worm phase. Indeed, TEM analysis (Fig. 2B) confirmed the desired highly anisotropic copolymer morphology and the digital photograph (see inset; tube inversion test) shows that a free-standing gel is formed at 20 °C. This worm gel was placed in a 4 °C fridge overnight to induce degelation and TEM studies indicated that a predominantly spherical morphology (plus a minor population of dimers/trimers) existed at this temperature. Allowing this cold, free-flowing liquid to return to 20 °C induced regelation. The thermoreversibility of this morphology transition was assessed by oscillatory rheology and small angle X-ray scattering (SAXS) studies (Fig. 2C and D). At 25 °C, the initial worm gel exhibited a *G*′ of 413 Pa, which was reduced to ≈0.2 Pa on cooling to 15 °C. Further cooling to 4 °C had no further effect on *G*′ and minimal hysteresis occurred when returning to 25 °C. However, a somewhat higher *G*′ of 614 Pa was determined for the reconstituted worm gel. SAXS experiments were performed on dilute, free-flowing aqueous dispersions of the as-synthesized and reconstituted PEG_57_-PHPMA_120_ worms. In principle, the low *q* gradient in an *I*(*q*) *vs.* (*q*) plot should be diagnostic of the predominant copolymer morphology,^52^ where *I*(*q*) is the X-ray scattering intensity and *q* is the scattering vector (*q* = 4π sin *θ*/*λ*). The low *q* gradient is close to −1 in both cases (see Fig. 2D; green and black open circles), which is characteristic of rod-like particles and also a good approximation for highly anisotropic block copolymer worms.^39^ Furthermore, satisfactory fits to these SAXS patterns could be obtained using a previously reported worm-like micelle model (see Fig. 2D; yellow and orange lines).^53^ Initially, the as-synthesized worms had a volume-average worm length (*L*_v_) of 1644 nm, a volume-average cross-sectional diameter (*D*_v_) of 28 nm and a mean Kuhn length of ∼140 nm. However, these worms become somewhat shorter and stiffer (and possibly also slightly thinner) after a 10% w/w copolymer worm gel was subjected to a thermal cycle prior to dilution to 1.0% w/w (*L*_v_ = 1328 nm, *D*_v_ = 26 nm, mean Kuhn length ∼170 nm). The greater Kuhn length calculated for the reconstituted worms is consistent with the higher gel modulus (*G*′) observed after the thermal cycle.^19^ A SAXS pattern was also recorded for the nanoparticles formed after cooling to 7 °C. This could be satisfactorily fitted using a previously reported ‘spheres, dimers and trimers’ model.^25^ As shown in Fig. 2D (see red data set), the low *q* gradient changed from approximately −1 to almost zero, suggesting a worm-to-sphere transition. More specifically, the data fit to the SAXS pattern recorded at 7 °C indicated a volume-weighted distribution of 47% spheres, 46% dimers and 7% trimers respectively. Hence the relatively large, highly anisotropic PEG_57_-PHPMA_120_ worms are converted into relatively small, approximately isotropic nanoparticles at 4 °C. This change in morphology accounts for the observed thermoreversible (de)gelation and is also sufficient to enable sterilization *via* cold ultrafiltration,^40^ which is essential for cell culture studies.

### Preparation of PEG_57_-PHPMA_65_ worm gels for stem cell studies

**Fig. 3:**
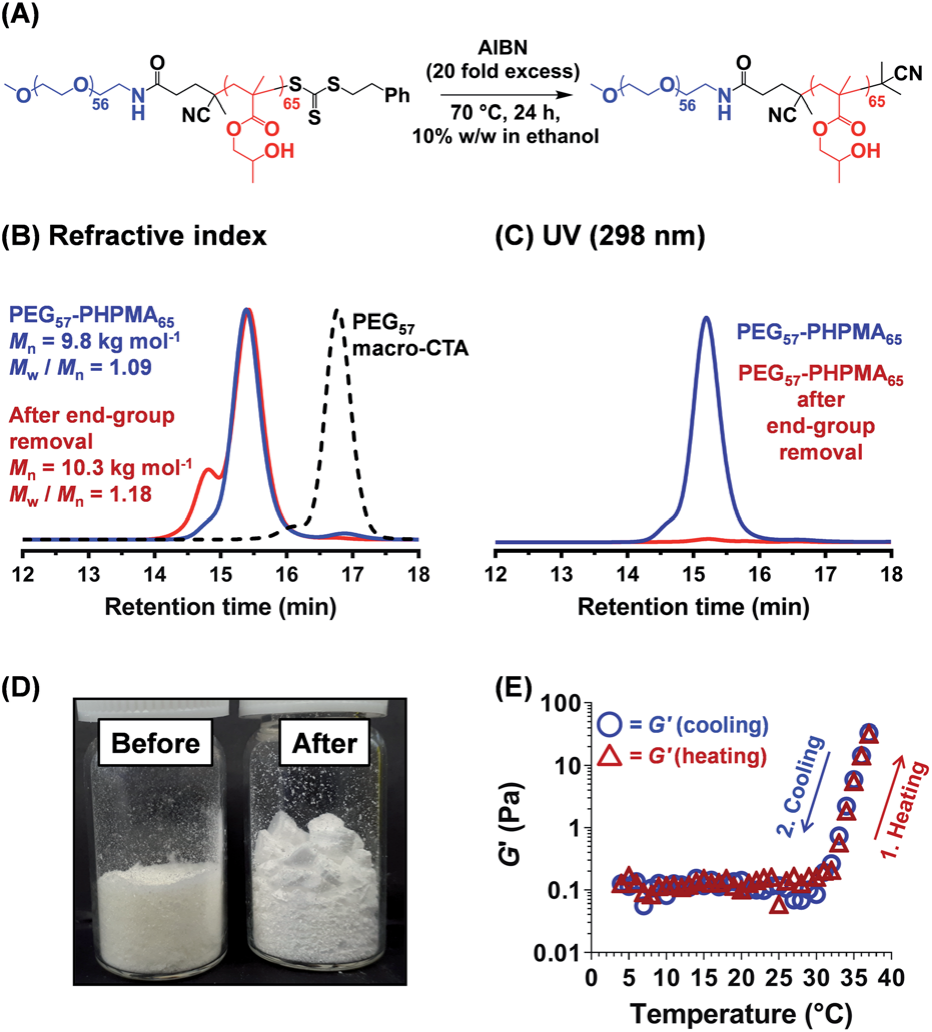
(A) Reaction scheme for the selective removal of the trithiocarbonate-based RAFT end-group from a PEG_57_-PHPMA_65_ diblock copolymer in an ethanol/water mixture by reaction with a twenty-fold excess of AIBN initiator (relative to the number of moles of end-groups). (B and C) GPC data recorded for this PEG_57_-PHPMA_65_ diblock copolymer using either a refractive index detector or a UV detector (*λ* = 298 nm). Analysis of the latter chromatograms suggests that 98% of the trithiocarbonate end-groups were removed. (D) Digital photographs recorded for as-synthesized and AIBN-treated PEG_57_-PHPMA_65_ powders indicate a subtle colour change from pale yellow to white after end-group removal. (E) Storage (*G*′) and loss moduli (*G*′′) obtained by oscillatory rheology during a 4 °C to 37 °C to 4 °C thermal cycle for a 12% w/w aqueous dispersion of PEG_57_-PHPMA_65_ nano-objects redispersed in a commercial cell culture medium (*Nutristem*). These data were obtained at 1 °C intervals using an angular frequency of 1.0 rad s^−1^ at an applied strain of 1.0%, allowing 2 min for thermal equilibration prior to each measurement.

Further optimization of the diblock copolymer composition was required to ensure that the rheological behavior observed for the salt-free aqueous worm dispersion at 25 °C (Fig. 2C and 3D) was also obtained for the reconstituted worms dispersed in the desired commercial cell culture medium (*Nutristem*) at 37 °C.^49^ This was achieved by lowering the PHPMA DP from 120 to 65. ^1^H NMR spectroscopy studies indicated a final HPMA conversion of more than 99%, with DMF GPC analysis indicating an *M*_n_ of 9.8 kg mol^−1^ and an *M*_w_/*M*_n_ of 1.09 (Fig. 3). It is well-known that organosulfur-based RAFT end-groups can be readily removed *via* various chemistries.^54–58^ In principle, removal of the trithiocarbonate end-groups should minimize malodor and perhaps also improve the biocompatibility of the worm gels.^59^ This was achieved by heating the PEG_57_-PHPMA_65_ diblock copolymer for 24 h in ethanol in the presence of a twenty-fold excess of AIBN initiator (Fig. 3).^54,60,61^ DMF GPC studies (refractive index detector) indicated a modest increase in both *M*_n_ and *M*_w_/*M*_n_, most likely owing to recombination of two PEG_57_-PHPMA_65_ radical chain-ends (Fig. 3B). More importantly, UV GPC analysis indicated that 98% of the trithiocarbonate end-groups were removed (Fig. 3C). Visual inspection of freeze-dried PEG_57_-PHPMA_65_ powder before and after end-group removal confirmed the expected color change from pale yellow to white (Fig. 3D). The AIBN-treated diblock copolymer was dialyzed against deionized water for 7 days at 4 °C, with dialyzate changes every 24 h. The aqueous dispersion was freeze-dried and reconstituted as a 12% w/w worm gel in a commercial stem cell culture medium (*Nutristem*).^62^ Temperature-dependent oscillatory rheological studies indicated a free-flowing liquid between 5 °C and 30 °C with a *G*′ of approximately 0.1 Pa, which is fully consistent with visual inspection (tube inversion test). However, further heating to 37 °C produced a rapid increase in *G′* up to 31 Pa and the formation of a free-standing gel, most likely owing to a sphere-to-worm transition. This thermal transition proved to be reversible with essentially the same free-flowing fluid being formed on cooling to 4 °C. In summary, a 12% w/w aqueous dispersion of PEG_57_-PHPMA_65_ in *Nutristem* exhibited the desired physical properties to proceed with the biological characterization and to test for stem cell stasis.

**Fig. 4:**
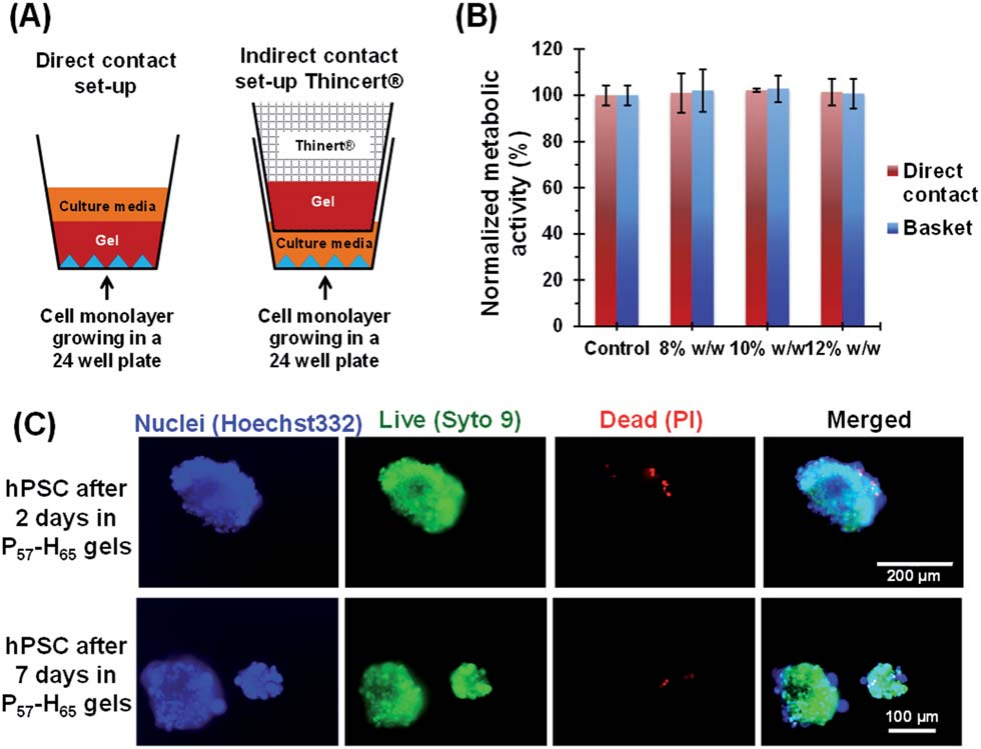
Biocompatibility assessment of a PEG_57_-PHPMA_65_ worm gel. (A) Schematic representation of the direct contact and indirect contact (ThinCert® inserts) set-ups employed to assess the toxicity of PEG_57_-PHPMA_65_ in the presence of human dermal fibroblast (HDF) cells. (B) Cell viability was evaluated by direct-contact cell monolayers and also *via* an indirect assay using ThinCert® inserts. Normalized HDF metabolic activity data obtained for triplicate experiments *via* MTT assays after exposure to the PEG_57_-PHPMA_65_ worm gel at a copolymer concentration of 8%, 10% or 12% w/w. Control experiments were performed to assess the metabolic activity of HDF cells in the absence of any PEG_57_-PHPMA_65_ copolymer. (C) Representative fluorescence microscopy images recorded for hPSC colonies after their immersion in a 12% w/w PEG_57_-PHPMA_65_ worm gel dispersed in *Nutristem* for either 2 or 7 days at 37 °C. Cell-permeable SYTO 9 (green fluorescent nucleic acid stain) and cell-impermeable propidium iodide (red fluorescent nucleic acid stain) were used for live/dead staining, respectively.

**Fig. 5:**
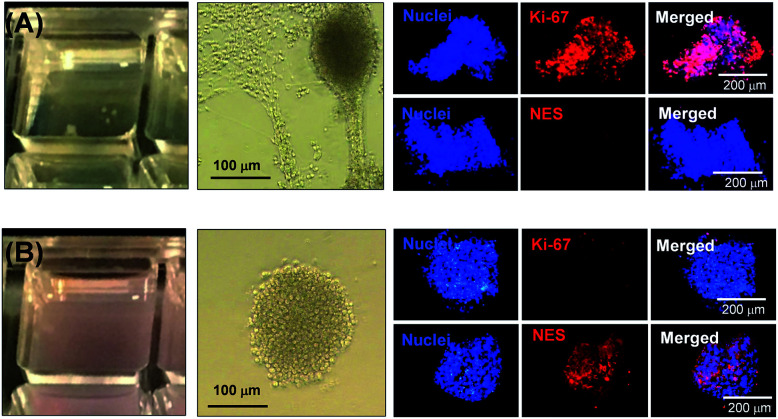
Digital photographs of hPSC colonies in culture media and optical microscopy images of the same colonies 7 days after their immersion in either a (A) 12% w/w PEG_57_-PHPMA_65_ or a (B) 6% w/w PGMA_55_-PHPMA_135_ worm gel. Fluorescence microscopy images of hPSC colonies obtained after degelation using and Ki-67 and NES immunolabeling. Colonies immersed in 12% w/w PEG_57_-PHPMA_65_ worm gel were Ki-67 (+) and NES (−), whereas colonies immersed in 6% w/w PGMA_55_-PHPMA_135_ worm gel gave Ki-67 (−) and NES (+). [N.B. In the former hydrogel, the cell culture medium (*Nutristem*) changes in colour from pink to yellow, indicating its gradual acidification. This is consistent with continuous cell proliferation within this hydrogel].

**Fig. 6:**
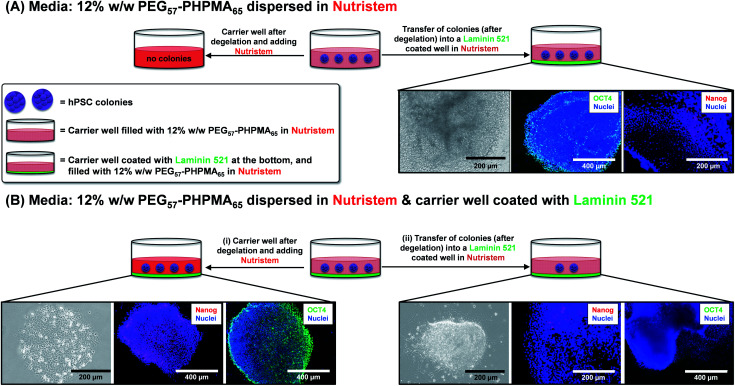
Immunolabeling of hPSC colonies after incubation in 12% w/w PEG_57_-PHPMA_65_ worm gels. (A) hPSC colonies were incubated in carrier wells for 7 days at 37 °C. Subsequent thermally-triggered degelation enabled these colonies to be transferred into carrier wells coated with Laminin 521. Colonies stained using OCT 4 and Nanog antibodies were analyzed *via* fluorescence microscopy. (B) The same hPSC colony incubation experiments were performed, but in this case Laminin 521 was coated on the bottom of the first carrier well. (i) Colonies that attached to Laminin 521 stained positive for OCT4 and Nanog markers and thus appear to remain pluripotent. (ii) In contrast, colonies that were recovered from the interior of the worm gel stained negative for OCT4/Nanog markers and thus are no longer pluripotent.

First, the biocompatibility of the cleaned PEG_57_-PHPMA_65_ was examined at copolymer concentrations of 8%, 10% and 12% w/w. A metabolic activity assay on human dermal fibroblasts (HDF) cells indicated that the PEG_57_-PHPMA_65_ worm gel was highly biocompatible at such copolymer concentrations (Fig. 4). Next, human pluripotent stem cell (hPSC) colonies were immersed in a 12% w/w PEG_57_-PHPMA_65_ worm gel in *Nutristem* for either 2 days or 7 days at 37 °C. Live/dead assays indicated that cell colonies remained viable for both time periods (Fig. 4C). Similar cell viability data were reported by Canton *et al.* for hPSC colonies immersed in a 6% w/w PGMA_55_-PHPMA_135_ worm gel [N.B. the difference in copolymer concentration for these two worm gels was necessary to achieve comparable gel strengths].^17^ However, the first clear indication that PEG_57_-PHPMA_65_ and PGMA_55_-PHPMA_135_ gels had a different effect on the hPSCs was a striking difference in the color of the cell culture medium over time. For hPSC colonies immersed in a 12% w/w PEG_57_-PHPMA_65_ worm gel, the cell culture medium invariably changed from pink to yellow over 7 days. For healthy sterile cell cultures, this color change typically indicates a shift to lower pH, which is consistent with proliferation and greater cell metabolic activity (Fig. 5A). In contrast, no color change was observed when using a 6% w/w PGMA_55_-PHPMA_135_ worm gel in *Nutristem*, which is known to induce stasis in such hPSC colonies (Fig. 5B).^17^ Furthermore, optical microscopy studies confirmed that the hPSC colonies immersed in the 12% w/w PEG_57_-PHPMA_65_ worm gel grew in size, with changes in colony morphology indicating cell proliferation (Fig. 5A). On the other hand, the morphology of hPSC colonies immersed in the 6% w/w PGMA_55_-PHPMA_135_ worm gel remained unchanged after 7 days (Fig. 5B), as previously reported.^17^ Next, we examined whether the hPSC colonies embedded in the PEG_57_-PHPMA_65_ worm gel were indeed proliferative; this was achieved by immunolabeling experiments. Accordingly, the PEG_57_-PHPMA_65_ worm gel was cooled to 4 °C to induce degelation and the isolated hPSC colonies were transferred to *Nutristem* within a new culture well that had been pre-coated with Laminin 521 (Fig. 6A).

**Fig. 7:**
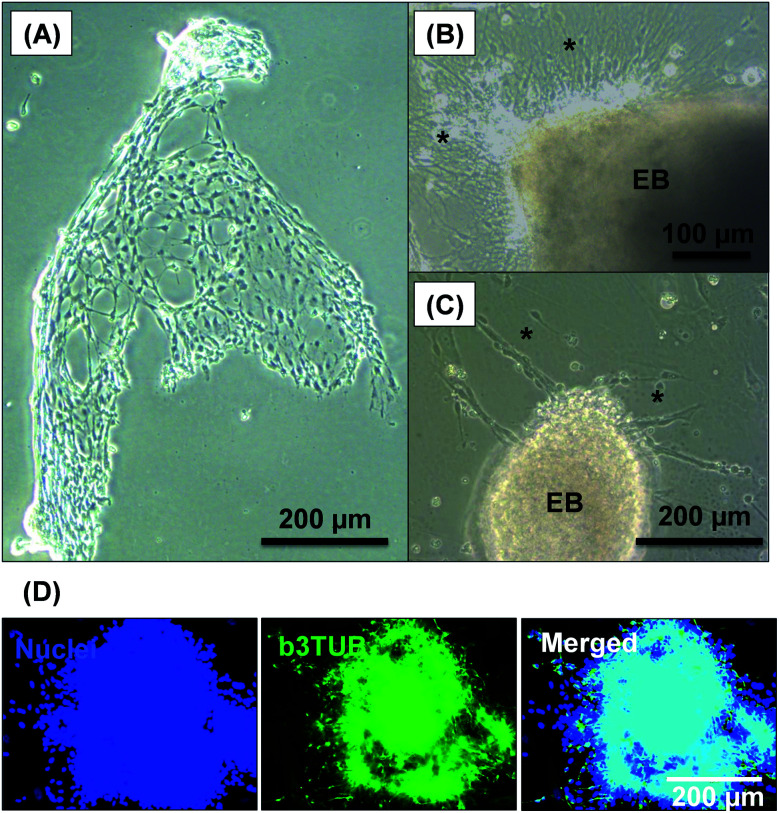
Optical microscopy images recorded for cell-like materials recovered following degelation of hPSC colonies after their immersion within PEG_57_-PHPMA_65_ worm gels for 7 days at 37 °C. (A) Such colonies form neural-like structures, where the asterisks shown in images (B) and (C) indicate neurons emerging from embryoid bodies (EB). (D) Immunolabeling of b3TUB revealed neural-like structures in the cell colonies and at the periphery of the EBs.

After removal from the worm gels, hPSC colonies were fixed and immunolabeled in turn with Ki-67 and nuclear envelope statin (NES). Ki-67 is a well-known marker for cell proliferation and is not expressed during the quiescent state (*G*_0_) of the cell cycle.^63–65^ On the other hand, NES is a specific marker for G_0_ and hence cell stasis.^66–68^ hPSC colonies immersed in the PEG_57_-PHPMA_65_ worm gel stained positive for Ki-67 and negative for NES. This immunoassay is consistent with our other observations (see above) and confirms beyond any doubt that such colonies continue to proliferate in this new hydrogel, rather than entering stasis (Fig. 5A). In contrast, hPSC colonies immersed in PGMA_55_-PHPMA_135_ worm gels stained negative for Ki-67 and positive for NES, confirming that this hydroxyl-functional hydrogel induces stasis (Fig. 5B).^17^ Overall, these observations suggest that a hydroxyl-rich environment is a prerequisite for hPSC colonies to enter the G_0_ state of the cell cycle. We also used OCT4 and Nanog as markers to explore whether the colonies embedded within the PEG_57_-PHPMA_65_ worm gel had retained their pluripotency. In this case, the hPSC stained negative for both OCT4 and Nanog, indicating loss of their original pluripotency and implying the onset of differentiation. Optical microscopy studies of hPSC colonies recovered after degelation revealed that they had differentiated to form neural-like cell colonies and embryoid bodies (EBs) (Fig. 7 and S3†). Such embryoid bodies often had an outermost area of growth in intimate contact with the gel that resembled neural-like cells. Further immunolabeling of these colonies using beta-3 tubulin antibody (b3TUB) produced a strong positive stain, and growth at the periphery of the recovered EBs indicates ectoderm and neural precursor differentiation.^69,70^ Human adult stem cells are mechanosensitive, and substrate rigidity is an *in vitro* extracellular switch that directs differentiation.^15,71^ Indeed, soft matrices that mimic brain tissue have been demonstrated to be neurogenic.^15^ Similarly, hPSCs are intrinsically mechanosensitive: both neural induction and caudalization can be accelerated by using a synthetic micro-engineered substrate comprising soft polydimethylsiloxane micropost arrays (PMAs).^72^ These results suggest that the PEG_57_-PHPMA_65_ worm gel can influence the differentiation of hPSC colonies. To examine this hypothesis, a series of further experiments were conducted in which hPSC colonies were immersed within PEG_57_-PHPMA_65_ worm gels placed in carrier wells, with each well bottom being coated with Laminin 521. After degelation, the colonies that had attached to the Laminin 521 remained pluripotent, as demonstrated by positive OCT4/Nanog immunoassays (Fig. 6B(i)). These observations suggest that PEG_57_-PHPMA_65_ worm gels can support the growth of pluripotent hPSC colonies provided that appropriate culture conditions to support pluripotency are maintained (*e.g.* the presence of Laminin 521, which is known to aid the proliferation of pluripotent stem cells).^73,74^ The hPSC colonies that did not attach to the Laminin 521 at the bottom of the well during gelation were isolated and placed in a new Laminin-coated well with *Nutristem*. Such colonies stained negative for OCT4/Nanog (Fig. 6B(ii)), indicating that they were no longer pluripotent. Presumably, the absence of any specific ECM ligand binding sites (*i.e.* Laminin 521) within the worm gel is responsible for this loss of pluripotency. With only a few exceptions, suspension cultures do not normally support the growth of hPSCs.^75–77^ Hence the behavior of hPSCs depends on the availability of suitable adhesive substrates. However, as hPSCs do not adhere to glass or conventional tissue culture plastics, specific ECM proteins, peptides, or synthetic polymers are required as culture substrates to retain their viability and pluripotency *in vitro*.^78^ Gradual loss of pluripotency has also been observed for hPSCs grown on uncoated microcarriers for continuous passaging.^78^ Thus, the lack of specific ligands within the PEG-PHPMA worm gel could indirectly influence differentiation of hPSC colonies immersed within such media.

## Conclusions

In summary, we have designed a new biocompatible PEG_57_-PHPMA_65_ worm gel that exhibits remarkably similar rheological behavior to that of a previously studied PGMA_55_-PHPMA_135_ worm gel.^17^ When pluripotent human stem cells are placed in the latter hydrogel they become non-proliferative, entering stasis within hours at 37 °C as previously reported.^42^ In striking contrast, immersing the same human stem cells within the PEG_57_-PHPMA_65_ worm gel does not induce stasis. This suggests that the hydroxyl-rich nature of the PGMA steric stabilizer expressed at the surface of the worms is essential for stasis induction. In this context, it is noteworthy that naturally-occurring mucins, which are believed to play an active role in the delayed gestation (diapause) for various mammalian embryos, also possess hydroxyl-rich chemical functionality. It is known that soft wholly synthetic substrates can induce stasis in somatic cells when used in conjunction with specific cell adhesion ligands.^79^ In the present study, both hydrogels are very soft (*G*′ = 10–50 Pa) and neither contain cell-specific binding motifs, which suggests that softness may be a necessary but not sufficient condition for stasis induction. Interestingly, addition of suitable adhesive substrates when immersing hPSCs within PEG_57_-PHPMA_65_ worm gels enabled the recovery of viable colonies. This suggests that the lack of adhesion motifs in such worm gels may influence stem cell differentiation. If this is correct, then in principle this effect could be modulated by incorporating relevant adhesion motifs within such gels. Hence, such gels may prove to be useful for the design of novel 3D biomimetic niches in tissue engineering applications. These new mechanistic insights are likely to have important implications for the design of new hydrogels that can induce stasis in embryonic stem cells. For example, other hydroxyl-based water-soluble polymers such as poly(2-hydroxypropyl methacrylamide), poly(2-hydroxyethyl acrylate), or poly(2-hydroxyethyl acrylamide) could be evaluated to determine whether there is a critical minimum level of hydroxyl functionality within the hydrogel that is required to induce stasis in hPSCs.

## Conflicts of interest

There are no conflicts to declare.

## Supplementary Material

SC-011-C9SC04734D-s001
