# Supplementary material for: Probing the mechanism for hydrogel-based stasis induction in human pluripotent stem cells: is the chemical functionality of the hydrogel important?
Source: Chem Sci. 2019 Nov 11;11(1):232–40. doi: 10.1039/c9sc04734d (PMC8133024; doi:10.1039/c9sc04734d)
Supplement: SC-011-C9SC04734D-s001 [file SC-011-C9SC04734D-s001.pdf]

Electronic Supplementary Material (ESI) for Chemical Science.
This journal is © The Royal Society of Chemistry 2019

Electronic Supporting Information for:

Probing the mechanism for hydrogel-based stasis induction in human pluripotent stem cells: is the chemical functionality of the hydrogel important?

M. Sponchioni, C. T. O'Brien, C. Borchers, E. Wang, M. N. Rivolta, N. J. W. Penfold,* I. Canton* and S. P. Armes*

Supplementary Figures

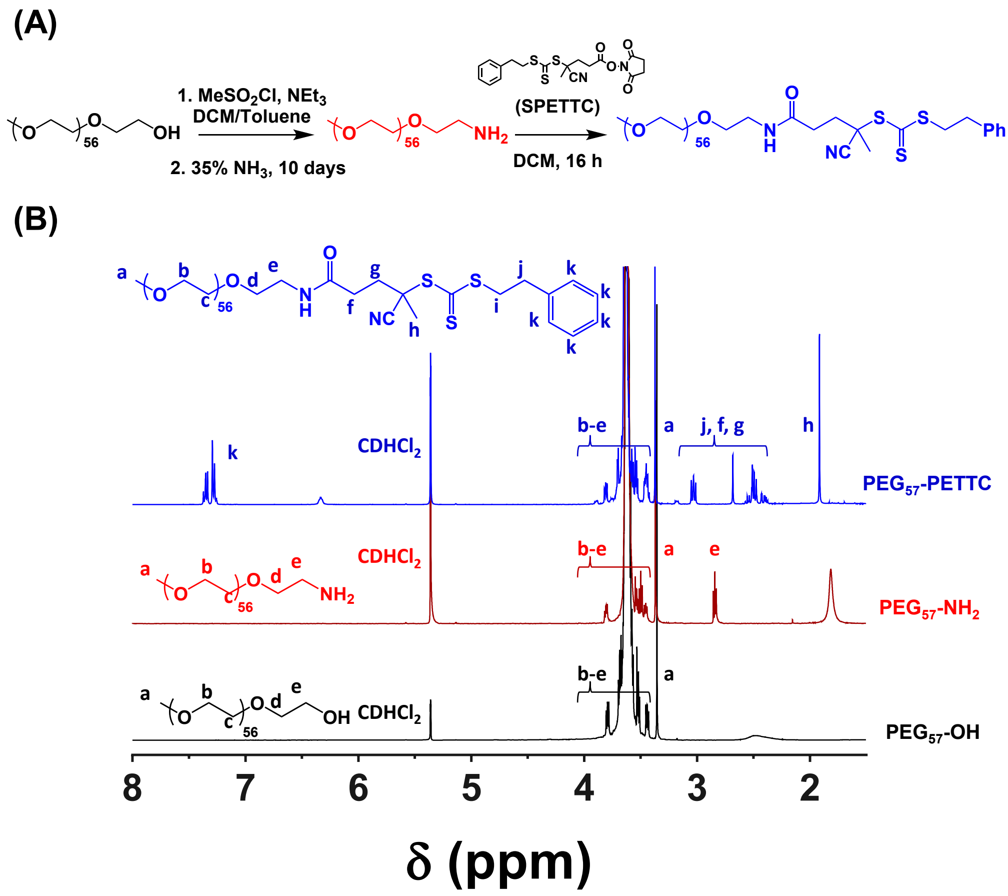

Figure S1. (A) Synthetic route for the preparation of the PEG_{57} macro-CTA used in this work and (B) corresponding ^1H NMR spectra. The monomethoxy precursor (PEG_{57}-OH, black spectrum) is functionalized to afford the corresponding monoamine (PEG_{57}-NH_2, red spectrum) and further reacted with SPETTC [see synthesis route in (A)) to yield the desired trithiocarbonate-based RAFT macro-CTA (PEG_{57}-PETTC, blue spectrum). ^1H NMR spectroscopy analysis indicated a degree of amidation of 93% by comparing the integrated aromatic proton signals at 7.2 – 7.4 ppm (see signal k, blue spectrum) to that of the PEG_{57} backbone protons (signals a-e) at 3.3 – 3.9 ppm.

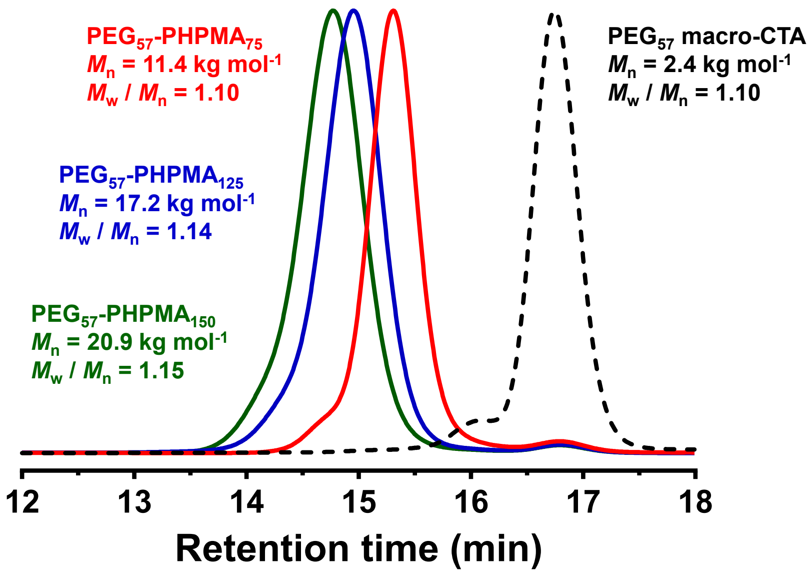

Figure S2. DMF GPC chromatograms obtained for the trithiocarbonate-based PEG_{57} macro-CTA and three PEG_{57}-$PHPMA_n$ diblock copolymers prepared at 15% w/w, where n = 75, 125 and 150. Molecular weight data are expressed relative to a series of near-monodisperse PEG standards.

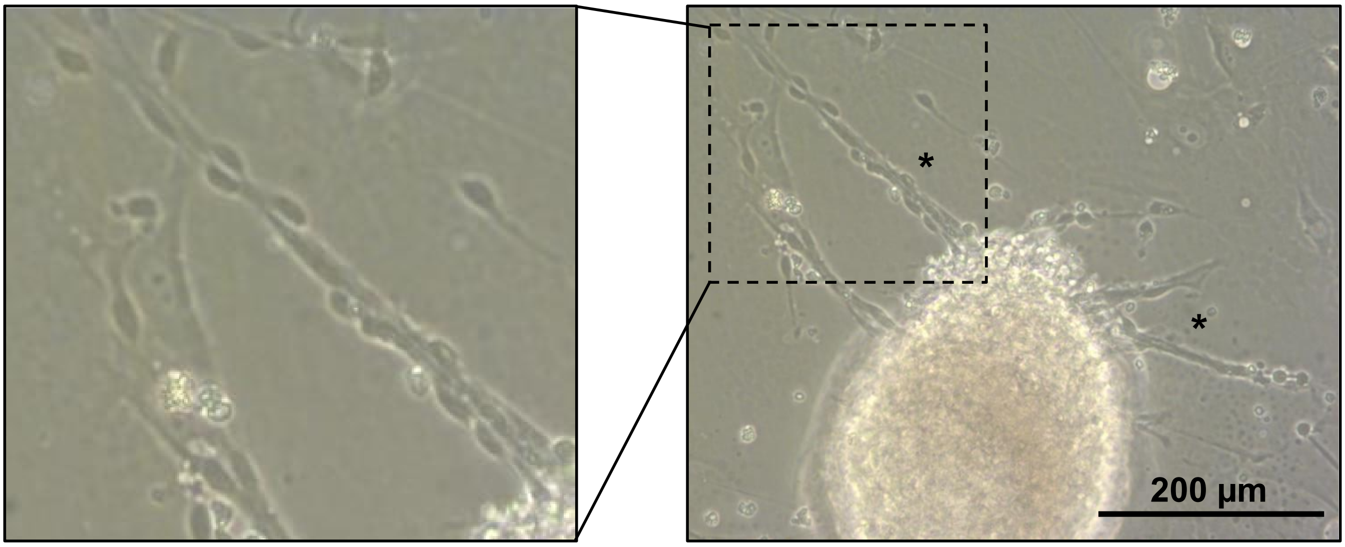

Figure S3. Optical microscopy images recorded for cell-like materials recovered following degelation of hPSC colonies after their immersion within PEG_{57}-$PHPMA_{65}$ worm gels for 7 days at 37 °C. These images are magnified versions of those shown in Fig 7B to aid visualization of the aligned neuron projections.

Supplementary Tables

Table S1. Summary of the monomer conversion, molecular weight distribution data and nanoparticle morphology (S = spheres, W = worms, V = vesicles, P = precipitate) for all the PEG_{57}-$PHPMA_x$ diblock copolymers prepared in this study.

Solids Concentration (% w/w)	Target PHPMA DP (*n*)	Conversion[a] (%)	M_n (kg mol^{-1})[b]	M_w / M_n[b]	Morphology[c]
5	75	>99	11.6	1.13	S
5	100	>99	16.1	1.12	S
5	110	>99	16.5	1.12	S&W
5	125	>99	17.3	1.11	S&W
5	150	>99	21.7	1.14	S&W
5	175	>99	23.5	1.14	V
5	200	>99	25.5	1.17	V
10	75	>99	11.4	1.09	S
10	100	>99	14.4	1.11	S
10	110	>99	15.3	1.12	S&W
10	125	>99	17.6	1.12	W
10	140	>99	18.1	1.12	W
10	150	>99	21.8	1.15	V
10	175	>99	23.4	1.16	V
10	200	>99	25.1	1.16	V
12.5	100	>99	15.8	1.12	S&W
12.5	110	>99	16.6	1.13	S&W
12.5	125	>99	17.9	1.13	W
15	75	>99	11.4	1.10	S
15	100	>99	15.4	1.12	S&W
15	110	>99	16.5	1.15	W
15	125	>99	17.2	1.14	W
15	140	>99	20.6	1.14	W
15	150	>99	20.9	1.15	V
15	175	>99	21.3	1.15	V
15	200	>99	22.1	1.16	P
20	75	>99	11.6	1.10	S
20	100	>99	14.3	1.12	S&W
20	110	>99	15.8	1.15	W
20	125	>99	17.4	1.14	W
20	140	>99	19.0	1.14	W
20	150	>99	19.8	1.15	V
20	175	>99	178.3	1.68	P
20	200	>99	329.5	2.52	P

Experimental Protocols

Materials

Poly(ethylene glycol) monomethyl ether (PEG_{57}-OH, M_n = 2650 g mol^{-1}), glycerol monomethacrylate (GMA) and 2-hydroxypropyl methacrylate (HPMA, mixture of isomers) were kindly donated by GEO Specialty Chemicals (Hythe, UK). Methanesulfonyl chloride (> 99%), triethylamine (> 99%), 4-4′azobis(4-cyanovaleric acid) (ACVA) and α,α′-azobisisobutyronitrile (AIBN, > 99%) were each purchased from Sigma Aldrich (UK) and used as received. 2,2′-Azobis[2-(2-imidazoline-2-yl)propane]dihydrochloride (VA-044) was purchased from Wako Pure Chemical Industries (Osaka, Japan) and used as received. Deionized water was obtained from an Elgastat Option 3A water purification unit with a resistivity of 15 MΩ cm. Anhydrous dichloromethane was collected from an in-house Grubbs purification system. All other chemicals or solvents (HPLC-grade) were purchased from either VWR, Sigma-Aldrich or Fisher and used as received. BioDesignDialysis Tubing (BID-050-030W) with a MWCO of 3500 was purchased from Fisher Scientific.

Cells

Maintenance and preparation of pluripotent stem cell lines

Human pluripotent stem cell (hPSC) lines, MasterShef (clinical grade) 14 and 7 were used. These were derived under licence from the HFEA and deposited with the UK Stem Cell Bank. hPSCs were maintained in human-feeder cultures using *Nutristem*® medium (Stemgent, UK) with non-enzymatic mechanical passage every 5 days.

Human dermal fibroblasts

Primary human dermal fibroblast (HDF) cells were obtained in batches from the ATCC, LGC standards (UK). Fibroblasts were routinely cultured in T75 flasks using standard culture medium (DMEM supplemented with 10% FCS, 2.0 mmol dm^{-3} L-glutamine, 0.625 mg dm^{-3} amphotericin B, 100 IU/ml penicillin and 100 mg dm^{-3} streptomycin). HDF cells were used for testing between passages 4 and 9. HDF cells were seeded at a density of 3 x 10^4 cells per well in a 24-well plate and cultured for 48 h prior to evaluation in standard culture media.

Synthesis of the PEG_{57} macro-CTA and the PEG_{57}-$PHPMA_n$ diblock copolymer nanoparticles

Synthesis of PEG_{57} macro-CTA

The synthesis of monoaminated PEG followed previously-published protocols (Figure S1).[1, 2] Monohydroxy-capped PEG_{57}-OH (26.5 g, 10.0 mmol, M_n = 2650 g mol^{-1}) was dissolved in toluene (500 mL) and this solution was distilled under a dry nitrogen atmosphere until approximately 200 mL remained. After cooling to room temperature, 200 mL of anhydrous dichloromethane was added, followed by dropwise addition of triethylamine (6.00 g, 59.3 mmol). Subsequently, methanesulfonyl chloride (6.79 g, 59.3 mmol) was added dropwise and the resulting reaction solution was stirred for 18 h under a nitrogen atmosphere. The insoluble triethylamine hydrochloride was removed by filtration and the organic solution was concentrated under vacuum before precipitation into excess diethyl ether. The white solid was collected by filtration and dried in a vacuum oven at 30 °C to yield PEG_{57}-OMs (20 g) which was subsequently dissolved in 32% aqueous ammonia (2 L) over 7 h. The lid was sealed and the solution was stirred at room temperature for 6 days. The lid was removed and the solution was stirred for a further 3 days to remove excess ammonia. The pH was then raised to 13 by adding NaOH (5 M) and the polymer was extracted using dichloromethane (3 x 250 mL). The organic phase was washed with brine, dried over magnesium sulfate and concentrated under reduced pressure. The product was then precipitated into excess diethyl ether and PEG_{57}-NH_2 was recovered by filtration and dried under vacuum. ^1H NMR spectroscopy confirmed 98% amination by comparing the integrated methoxy end-group at 3.3 – 3.4 ppm to that of the triplet assigned to the α-CH_2 of the amine group to triplet at 2.7 – 2.9 ppm (Figure S1). The succinimide-functionalized RAFT agent, SPETTC, was synthesized as described previously[2] and reacted with PEG_{57}-NH_2. All glassware was dried at 150 °C overnight and flame-dried under vacuum before use. SPETTC (1.90 g, 4.3 mmol) was dissolved in anhydrous dichloromethane (10 mL) in a 250 mL round-bottomed flask equipped with a pressure-equalizing dropping funnel. PEG_{57}-NH_2 (10 g, 3.8 mmol) was dissolved in anhydrous dichloromethane (70 mL) and added to the dropping funnel *via* cannula transfer under dry nitrogen. The PEG_{57}-NH_2 solution was added dropwise to the SPETTC solution over 1 h and then stirred for 16 h at 20 °C. The crude product was precipitated three times into excess diethyl ether, collected by vacuum filtration

and finally dried in a vacuum oven at 30 °C to yield the desired trithiocarbonate-capped PEG_{57} macro-CTA. ^{1}H NMR spectroscopy was used to calculate a degree of amidation of 93% by compariing the integrated oxyethylene protons associated with the PEG backbone at 3.3 – 4.0 ppm to that of the integrated aromatic end-group signal at 7.2 – 7.4 ppm. DMF GPC studies indicated an M_n of 2.4 kg mol^{-1} and an M_w / M_n of 1.10 (using a series of near-monodisperse PEG standards).

Synthesis of PEG_{57}-$PHPMA_n$ diblock copolymer nano-objects by RAFT aqueous dispersion polymerization of HPMA

The synthesis protocol used for the PEG_{57}-$PHPMA_{125}$ diblock copolymer nano-objects at 10% w/w is representative. A 14 mL glass vial was charged with a magnetic flea, PEG_{57} macro-CTA (0.1100 g, 34.5 µmol), HPMA monomer (0.62 g, 4.30 mmol, target DP = 125), VA-044 (3.70 mg, 11.4 µmol) and deionized water (6.6 g) to afford a 10% w/w yellow solution. The sealed reaction vessel was degassed with dry nitrogen in an ice/water mixture for 20 min and then placed in a preheated oil bath set at 40 °C for 4 h. The polymerization was quenched by exposing the reaction solution to air and cooling to room temperature. A series of PEG_{57}-$PHPMA_n$ diblock copolymer nano-objects were prepared by systematically varying the target DP of the PHPMA block (n) and the copolymer concentration (see Table S1). Block copolymer dispersions were assessed by ^{1}H NMR spectroscopy to examine monomer conversion, DMF GPC to determine the molecular weight distribution and TEM to determine the copolymer morphology. Where appropriate, temperature-dependent rheology studies were conducted on selected worm dispersions in either water or cell culture medium (Nutristem).

Preparation of PEG_{57}-$PHPMA_{65}$ worm gels for cell culture experiments

Synthesis of PEG_{57}-$PHPMA_{65}$ worms

A 250 mL round bottomed flask was charged with PEG_{57} macro-CTA (93% functionality, 1.602 g, 502 µmol), HPMA (4.680 g, 32.5 mmol, target DP = 65), VA-044 (0.0530 g, 164 µmol, [PEG_{57} macro-CTA] / [VA-044] = 3.0) and water (56.9 g) to afford a 10% w/w yellow solution. The reaction vial was fitted with a suba-seal, placed in an ice/water bath and degassed using N_2 for 30 min, and then immersed in a preheated oil bath set at 56 °C for 4 h. The polymerization was quenched by exposing the reaction solution to air and cooling to room temperature. ^{1}H NMR spectroscopy studies confirmed more than 99% HPMA conversion, while DMF GPC analysis indicated an M_n of 9,800 g mol^{-1} and an M_w / M_n of 1.09 (expressed relative to a series of near-monodisperse PEG standards).

Removal of RAFT chain-ends and purification by dialysis prior to cell studies

An as-synthesized 10% w/w aqueous dispersion of PEG_{57}-$PHPMA_{65}$ worms was freeze-dried to give a pale yellow powder. A 250 mL round-bottomed flask was charged with PEG_{57}-$PHPMA_{65}$ diblock copolymer powder (6.305 g, 509 µmol) and ethanol (55.0 g) to afford a 10% w/w pale yellow ethanolic solution. AIBN (1.673 g, 10.1 mmol; [AIBN] / [PEG_{57}-$PHPMA_{65}$] = 20) was added and the heterogeneous solution was sealed and degassed with dry nitrogen for 30 min using an ice/water mixture. The round-bottomed flask was then placed in a preheated oil bath at 70 °C for 24 h. The reaction was quenched by exposing the solution to air and cooling to room temperature. The copolymer solution was concentrated under reduced pressure, precipitated into a ten-fold excess diethyl ether at 0 °C and isolated via vacuum filtration to give a white powder. This white powder was dissolved in deionized water (35 g) at 4 °C to give a 15% w/w aqueous solution and then placed on a rotary evaporator to remove residual diethyl ether. Prior to biocompatibility and stem cell stasis experiments, any small molecule impurities were removed via dialysis (MWCO = 3500) against water for 7 days at 4 °C, with dialysates being changed approximately every 12 h. The purified aqueous copolymer solution was then freeze-dried to afford a fine white powder. Almost complete removal of the trithiocarbonate chain-ends (98%) was confirmed by UV-GPC studies (tuned to the absorption maximum of 298 nm for the trithiocarbonate end-groups). The M_n increased slightly to 10.3 kg mol^{-1} while the final M_w/M_n was 1.18. This purified PEG_{57}-$PHPMA_{65}$ was redispersed in cell culture media (*Nutristem*) at 12% w/w. The temperature was maintained at approximately 4°C using an ice bath and magnetic stirring was continued for at least 20 min until full dispersion was achieved, then this free-flowing aqueous worm dispersion was used as required.

Preparation of $PGMA_{55}$-$PHPMA_{135}$ worm gels for cell culture experiments

Synthesis of the $PGMA_{55}$ macro-CTA

CPDB (0.80 g, 3.6 mmol) and glycerol monomethacrylate (GMA, 40.59 g, 0.25 mol) were weighed into a 250 ml round-bottomed flask and purged with dry nitrogen for 20 min. ACVA (202.9 mg, 0.72 mmol) was added and the reaction solution was degassed for a further 5 min. Degassed anhydrous ethanol (61 mL) was added and the solution was again degassed for a further 5 min prior to immersion in an oil bath set at 70°C. After 2 h, a ^{1}H NMR spectrum recorded in CD_3OD indicated approximately 80 % GMA monomer conversion. The crude polymer was purified by precipitating twice into excess dichloromethane from methanol to remove unreacted monomer. Then the polymer was isolated via filtration and the resulting solid was dissolved in water (200 mL). Residual dichloromethane was evaporated at 30°C using a rotary evaporator. Once all traces of solvent were removed, the aqueous solution was freeze-dried overnight to afford a pink powder. ^{1}H NMR spectroscopy studies of the purified polymer dissolved in CD_3OD indicated a mean degree of polymerization of 55. DMF GPC analysis indicated an M_n of 14,100 g mol^{-1} and an M_w/M_n of 1.09 (expressed relative to a series of near-monodisperse poly(methyl methacrylate) standards for calibration).

Synthesis of PGMA-PHPMA diblock copolymers worms in 0.15 M PBS at 20% w/w solids

$PGMA_{55}$ (3.023 g, 0.33 mmol) and HPMA (6.240 g, 41.62 mmol) were weighed in turn into a 100 mL round-bottomed flask and purged with dry nitrogen for 20 min. ACVA (31.0 mg, 0.11 mmol) was added and the flask was degassed for a further 5 min. Phosphate-buffered saline (PBS) solution (Dulbecco A, Oxoid, Basingstoke, 37 mL, 150 mM, previously purged with N_2 for 30 min) was then added and the solution was degassed for a further 5 min prior to immersion of the reaction flask in an oil bath set at 70°C for 2 h, after which ^{1}H NMR spectra recorded in CD_3OD indicated almost 100 % HPMA conversion (as judged by integration of the attenuated vinyl signals at 5.6 and 6.2 ppm). DMF GPC analysis indicated an M_n of 35,900 g mol^{-1} and an M_w/M_n = 1.10 (expressed relative to a series of near-monodisperse poly(methyl methacrylate) standards).

Dialysis of PGMA-PHPMA worm gels prior to cell culture experiments

The as-synthesized 20% w/w $PGMA_{55}$-$PHPMA_{135}$ gel was dialyzed against pure water for seven days at 4 °C, with dialysate changes every 12 h (MWCO = 1,000). The resulting aqueous dispersion was freeze-dried to yield a fine pink powder, which was redispersed in cell culture media (*Nutristem*) at 6% w/w. The temperature was maintained at approximately 4°C using an ice bath and magnetic stirring was continued for at least 20 min until full dispersion was achieved.

Sterilization protocol for $PGMA_{55}$-$PHPMA_{135}$ and PEG_{57}-$PHPMA_{65}$ block copolymer nanoparticles prior to cell culture experiments

The 12% w/w aqueous dispersions of PEG_{57}-$PHPMA_{65}$ (and 6% w/w $PGMA_{55}$-$PHPMA_{135}$) worm gel in the chosen cell culture medium were cooled to 4°C to induce a worm-to-sphere transition and hence subsequent degelation to afford a free-flowing dispersion of copolymer spheres. This cold, low-viscosity fluid was then ultrafiltered using a sterile 0.20 µm syringe filter into a sterile vessel within a laminar flow cabinet. Syringes and filters were stored at -20°C for at least 1 h prior to ultrafiltration to prevent gelation on contact. The resulting sterilized copolymer dispersion was then used immediately for cell colony encapsulation experiments or stored at either 4°C or -20°C for future use (depending on the specifications of the cell medium).

Cell viability in direct contact with PEG_{57}-$PHPMA_{65}$ gels

Cell viability assays on human dermal fibroblasts (HDFs) using MTT assay

HDFs were seeded in 24-well plates at a density of 3 x 10^4 cells per well and grown until 80% confluence (typically 48 h). Gels were evaluated both in direct contact with the cells and also in non-direct contact (basket method). A non-contact *ThinCert*® (Greiner Bio-One, UK) set-up was used to identify any toxic low molecular weight compounds that might be present in the worm gels (e.g. unreacted HPMA monomer). *ThinCert*® comprises small baskets of tissue culture plastic with a polycarbonate membrane bottom that fits over a 24-well plate. Thus cells were exposed to the gel through the cell medium in the 24-well plates, but not by direct contact. This set-up discriminates between the effect of direct contact of the worm gel on the cells and the effect of residual small molecule impurities. For the indirect contact set-up, 250 µL of the 12% w/w copolymer gel was added to each *ThinCert*® basket. Cells were placed below each basket and immersed in the appropriate cell culture medium (500

μL). For the direct contact set-up, the cell medium was removed from the wells and the gel (typically 500 μL) was applied directly onto the cell monolayers. Gel batches were tested on 80% confluent HDF cells over 24 h. Cell viabilities were then assessed *via* an MTT assay (3-(4,5-dimethylthiazol-2-yl)-2,5 diphenyl tetrazolium bromide) (Sigma-Aldrich, St Louis, MO). Briefly, cells were washed at 4°C with cold PBS, then incubated with MTT solution (0.50 g dm^{-3} MTT in PBS at 20 °C, 1 mL per well of a 24-well plate) for 1 h at 37°C in a humidified incubator (5% CO_2/95% air). In healthy viable cells, MTT is reduced to a purple formazan salt by the mitochondrial enzyme, succinyl dehydrogenase, which allows spectrophotometric quantification of cell viability. After 1 h, the solution was aspirated and the insoluble intracellular formazan product was solubilized and removed from cells by adding acidified iso-propanol (0.30 mL per well of a 24-well plate), followed by incubation for 10 min. The absorbance at 540 nm was then determined using a plate-reading visible absorption spectrophotometer, with the absorbance at 630 nm being used as a reference. Mean viability data and SEM were normalized using a negative control (no treatment, 100% viability) and expressed as a percentage viability ± SEM. Experiments were performed in duplicate well samples with n = 3 independent experiments. For statistical analysis, the student's paired t-test was used for the raw data to assess the significance of differences between the samples and the control group.

Gelation of hPSC colonies

The hPS cells were typically grown using *Nutristem*® medium (Stemgent, UK) in human feeders, unless otherwise stated. When cultures achieved optimal cell density (typically 60-70% surface coverage), the cell medium was replenished and colonies were mechanically harvested. Colonies were placed onto 35 mm Petri dishes in preparation for gel seeding onto Ibidi eight-well slides with or without Laminin 521 coating (Biolamina, Sweden). Such slides were placed on ice and 500 □L of a cold 6% $PGMA_{55}$-$PHMPA_{135}$ or 12% w/w PEG_{57}-$PHPMA_{65}$ copolymer dispersion (which is a free-flowing liquid at ~ 4°C) was added to each of the wells. Using a sterile plastic Pasteur pipet equipped with a 'super-fine' tip, individual colonies were placed on the center of each ibidi well and gently stirred to allow mixing. Gelation was immediately triggered by placing the ibidi wells in a humidified incubator (5% CO_2/95% air) set at 37°C for the desired time period (i.e. 2 or 7 days) prior to harvesting by degelation. Degelation was triggered by placing each ibidi slide on ice for approximately 5 min. The resulting free-flowing copolymer containing the cell colonies was diluted ten-fold with Nutristem® (5.0 mL) into a Laminin 521-coated six-well plate releasing the cell colonies. Additionally, the Ibidi eight-well slides (with and without Laminin 521 coating) used for the gelation were not discarded but instead replenished with 500 □l of medium and inspected by optical microscopy for signs of hPSC colony growth. The six-well plates were stored for approximately 3 h in a humidified incubator (5% CO_2/95% air) to allow viable cell colonies to adhere to the matrix. Subsequently, media were replenished daily.

Live/Dead cell assay

The viabilities of hPSC colonies immersed within PEG_{57}-$PHPMA_{65}$ worm gels were assessed using a commercial live/dead assay (Life Technologies, UK). This assay utilizes a binary mixture of a cell-permeable SYTO® 9 green fluorescent nucleic acid stain (excitation at 480 nm, emission at 500 nm) and an impermeable red fluorescent nucleic acid stain, propidium iodide (PI, excitation at 490 nm, emission at 635 nm). Cells with compromised (i.e. leaky) membranes are designated as dead or dying and are stained red (PI), whereas cells with intact membranes are stained green (SYTO® 9). When used alone, the latter stain generally labels all cells, but when both dyes are present the PI penetrates damaged membranes and quenches the green fluorescence due to SYTO® 9, so that this signal is not detected. Briefly, the gelled colonies were cooled to around 4°C for 5 min to trigger degelation and then allowed to sediment under gravity. The free-flowing aqueous copolymer dispersion supernatant was partially removed and colonies were washed once with cell culture medium pre-cooled to 4°C. The aqueous fluid was then removed and warm (37°C) cell culture medium was added to each well containing SYTO® 9 (15 □M) and PI (60 □M). Cells were incubated in a humidified incubator (5% CO_2/95% air) for 25 min in order to allow dye uptake to occur. Then cell nuclei were counter-stained for a further 5 min with Hoechst 33342 (Life Technologies, UK). Finally, colonies were washed with PBS (pre-cooled to 4°C) and further culture medium (depending on the vessel, typically 3 mL for a six-well plate and 500 □L for ibidi imaging plates) was added prior to inspection using an EVOS® epifluorescence imaging system.

Evidence for stasis (suspended animation):

Ki-67/nuclear envelop statin immunolabeling experiments

Colonies were isolated from the worm gels by incubation on ice for approximately 5 min to induce degelation. Each well was then collected into 1.5 mL Eppendorf tubes containing ice-cold PBS (1 mL). Colonies were washed twice at 4°C for 5 min (1000 rcf) and then fixed for 30 min using an aqueous solution of 4% formaldehyde in PBS (100 □L). Control colonies (not gelled) and gel-recovered colonies growing in six-well plates were also washed in cold PBS and fixed with 4% formaldehyde in PBS. All samples were then washed three times in PBS and permeabilized using a 0.1% Triton X100 PBS solution for 20 min (1 mL per well in a six-well plate and 100 □L per Eppendorf

tube). Colonies were then washed three times in PBS and blocked in 5% BSA-PBS for 2 h at 20°C, prior to incubation with a primary antibody solution (1:100 rabbit anti-human Ki-67 monoclonal antibody (Abcam) + 1% BSA in PBS; 1:20 mouse anti-human S-44 nuclear statin antibody 1% BSA in PBS) overnight at 4°C with gentle rocking. These antibody-labeled colonies were then washed three times in PBS and then incubated with a secondary antibody solution (1:1000 Goat anti-rabbit Cy3 IgG (Abcam) + 1% BSA in PBS; 1:1000 Goat anti-mouse Chromeo® 546 (Abcam) + 1% BSA in PBS)[3] for 1 h at 20°C with gentle rocking. Colonies were washed three times with PBS and cell nuclei were counter-stained for 5 min using Hoechst 33342 (Life Technologies, UK). Finally, each sample was washed three times in PBS prior to inspection using an EVOS® epifluorescence imaging system.

Immunolabeling experiments

Oct-4/Nanog and b-TUB immunolabeling experiments

Colonies recovered from gels were allowed to attach to Laminin 521-coated 6-well plates for up to 48 h. Colonies were then washed with PBS and fixed for 30 min using an aqueous solution of 4% formaldehyde in PBS (100 □L). All samples were then washed three times in PBS and permeabilized using a 0.1% Triton X100 PBS solution for 20 min. Colonies were then washed three times in PBS and blocked in 5% BSA-PBS for 2 h at 20°C, prior to incubation with a primary antibody solution (1:100 rabbit anti-human Oct-4 antibody (Abcam, UK) + 1% BSA in PBS; 1:100 rabbit anti-human Nanog (Cell Signaling Technology, USA) 1% BSA in PBS); (1:100 rabbit anti-human beta-Tubulin antibody (Abcam, UK) overnight at 4°C with gentle rocking. These antibody-labeled colonies were washed three times in PBS and then incubated with a secondary antibody solution (1:1000 Goat anti-rabbit Alexa Fluor® 488 IgG (Abcam) + 1% BSA in PBS; 1:1000 Mouse anti-rabbit Cy3 IgG (Abcam) + 1% BSA in PBS); (1:1000 Goat anti-rabbit Alexa Fluor® 488 IgG (Abcam) for 1 h at 20°C with gentle rocking. Colonies were washed three times with PBS and cell nuclei were counter-stained for 5 min using Hoechst 33342 (Life Technologies, UK). Finally, each sample was washed three times using PBS prior to inspection using an EVOS® epifluorescence imaging system.

Chemical Characterization

^{1}H NMR Spectroscopy

All ^{1}H NMR spectra were recorded using a 400 MHz Bruker Avance-400 spectrometer operating at 298 K with 16 scans averaged per spectrum. Spectra for all RAFT agents and PEG_{57} precursors were recorded in CD_2Cl_2, whereas those for all diblock copolymers were recorded in CD_3OD.

Small Angle X-ray Scattering (SAXS)

SAXS patterns were recorded at a synchrotron source (Diamond Light Source, station I22, Didcot, UK) using monochromatic X-ray radiation (wavelength λ = 0.124 nm, with q ranging from 0.015 to 1.3 nm^{-1}, where $q = 4\pi \sin \theta/\lambda$ is the length of the scattering vector and θ is one-half of the scattering angle) and a 2D Pilatus 2M pixel detector (Dectris, Switzerland). Measurements were conducted on 1.0% w/w aqueous dispersions and at approximately 25 °C and 7 °C. X-ray scattering data were reduced and normalized using standard routines by the beamline. Modeling was performed using Irene SAS Pro software.

Worm model

The worm-like micelle form factor in equation S1 is expressed as:[4]

$$F_{w\_mic}(q) = N_w^2\beta_s^2F_{sw}(q) + N_w\beta_c^2F_c(q,R_g) + N_w(N_w - 1)\beta_c^2S_{cc}(q) + 2N_w^2\beta_s\beta_cS_{sc}(q) \quad \text{(S1)}$$

where the core block and the corona block X-ray scattering length contrast are given by $\beta_s = V_s(\xi_s - \xi_{sol})$ and $\beta_c = V_c(\xi_c - \xi_{sol})$, respectively. Here, ξ_s, ξ_c and ξ_{sol} are the X-ray scattering length densities of the core block (ξ_{PHPMA} = 12.21 x 10^{10} cm^{-2}), the corona block (ξ_{PEG} = 10.41 x 10^{10} cm^{-2}) and the solvent (ξ_{sol} = 9.39 x 10^{10} cm^{-2}) respectively and V_s and V_c are the volumes of the core block (V_{PDMA}) and the corona block (V_{PDMS}) respectively. The volumes

were calculated from $V = \frac{M_{n,poly}}{N_A \rho}$ using the known density of PHPMA (ρ_{PHPMA} = 1.33 g cm^{-3}) and the known density of PEG (ρ_{PEG} = 1.13 g cm^{-3}), where $M_{n,pol}$ corresponds to the number-average molecular weight of the block determined by ^{1}H NMR spectroscopy. The self-correlation time for the worm-like micelle core of radius R_{sw} is:

$$F_{sw}(q) = F_{worm}(q,L_w,b_w) A_{cs_{worm}}^{\ 2}(q,R_{sw}) \qquad \text{(S2)}$$

This is a product of a core cross-section term:

$$F_{csworm}(q,R_g) = A_{cs_{worm}}^{\ 2}(q,R_s) = \left[2\frac{J_1(qR_{sw})}{qR_{sw}} \right]^2 \qquad \text{(S3)}$$

where J_1 is the first-order Bessel function of the first kind, and a form factor $F_{worm}(q,L_w,b_w)$ for self-avoiding semi-flexible chains represents the worm-like micelle, where b_w is the worm Kuhn length and L_w is the mean worm contour length. A complete expression for the chain form factor can be found elsewhere.[5] The self-correlation term for the corona block is given by the Debye function shown in equation S3. The interference cross-term between the worm micelle core and the corona chain is given by:

$$S_{sc}(q) = \Psi^2(qR_g) J_0^2[q(R_{sw} + R_g)] F_{worm}(q,L_w,b_w) \qquad \text{(S4)}$$

where $\Psi(qR_g) = \frac{1 - exp^{\left(-q^2R_g^2\right)}}{(qR_g)^2}$ is the form factor amplitude of the corona chain, R_g is the radius of gyration of the PEG corona block and J_0 is the zero-order Bessel function of the first kind. The interference term between the worm corona chains is:

$$S_{cc}(q) = \Psi(qR_g) A_{cs_{worm}} J_0[q(R_{sw} + R_g)] F_{worm}(q,L_w,b_w) \qquad \text{(S5)}$$

Spheres, dimers and trimers model

The total scattering intensity for a mixture of spherical spheres, dimers, and trimers, *I*, can be expressed as:

$$I = k_1 \Phi^2(qR_{ss}) + \Phi^2(qR_{ss}) \sum_{n=2}^{3} n k_n S_n(q) + c_c F_c(q,R_g) \qquad \text{(S6)}$$

where n is the number of spheres forming unimers (n = 1), dimers (n = 2) or trimers (n = 3), and k_n is the volume fraction of each nano-object in the sample, $\boldsymbol{\Sigma}_{n=1}^{3}$, $k_n = 1$. $\Phi(qR_{ss}) = 3[\sin(qR_{ss}) - qR_{ss}\cos(qR_{ss})]/(qR_{ss})^3$ is the form factor amplitude of a sphere of radius R_{ss}. The second term in equation S6 represents the form factor for spherical dimers and trimers, where $S_n(q)$ can be obtained using the Debye equation:[6]

$$S_n(q) = 1 + \frac{2}{n} \sum_{i=1}^{n-1} \sum_{i=i+1}^{n} \frac{\sin(qr_{ij})}{qr_{ij}} \qquad \text{(S7)}$$

and the inter-sphere separation distances are expressed as $r_{12} = r_{23}$ and $r_{13} = 4R_{ss}$. The background scattering of the PEG corona block is modeled using the Debye function, $F_c(q,R_g) = 2[exp(-q^2R_g^2) - 1 + q^2R_g^2]/(q^4R_g^4)$. The radius of gyration for the corona block is R_g and c_c is the relative concentration of the corona block. These five parameters

(R_{ss}, R_g, c_c, k_2 and k_3) are used to fit the SAXS data. Programming tools within the Irena SAS Igor Pro macros[7] were used for model fitting.

The fitted R_g was about 2.1 nm, which is close to the calculated value. Assuming that the projected contour length of an ethylene glycol repeat unit is 0.37 nm (estimated from the crystal structure of PEG homopolymer),[8] the contour length of the PEG_{57} corona block is 21.1 nm (57 x 0.37). If the PEG Kuhn length is 1.0 nm,[9] then the radius of gyration of an unperturbed PEG chain is $(21.1/6)^{0.5}$ = 1.87 nm.

Transmission Electron Microscopy (TEM)

Aqueous block copolymer dispersions were diluted from 10% w/w to 2% w/w with deionized water and gently stirred overnight at room temperature. These dispersions were further diluted to 0.1% w/w and stirred for a further 3 h. Copper/palladium grids were surface-coated in-house to produce a thin film of carbon, which were then plasma glow-discharged for 30 seconds to give a hydrophilic surface. A 10 µL droplet of 0.1% w/w aqueous dispersion was placed on the hydrophilic carbon/palladium grid for 40 seconds, blotted to remove excess sample and then negatively stained with uranyl formate solution (0.75% w/w, 10 µL) for a further 20 seconds. Excess stain was removed by blotting with filter paper and carefully dried using a vacuum house. Imaging was performed using a FEI Tecnai Spirit 2 microscope operating at 80 kV and equipped with an Orius SC1000B camera.

Gel Permeation Chromatography (GPC)

Aqueous copolymer dispersions were freeze-dried overnight to obtain pale yellow powders. 0.50% w/w copolymer solutions were prepared in HPLC-grade DMF containing 10 mM LiBr and DMSO (1.0 % v/v) was used as a flow rate marker. GPC studies were conducted at 60 °C using a flow rate of 1.0 mL min^{-1}. The GPC set-up comprised an Agilent 1260 Infinity series degasser and pump, an Agilent PL-gel guard column, two Agilent PL-gel Mixed-C columns, a RI detector and a UV detector set at a wavelength of 298 nm. Nine near-monodisperse poly(ethylene glycol) standards with M_p values ranging from 599 g mol^{-1} to 969,000 g mol^{-1} or eleven near-monodisperse poly(methyl methacrylate) standards with M_p values ranging from 2380 g mol^{-1} to 2,200,000 g mol^{-1} were used for calibration.

Rheology Studies

An AR-G2 rheometer equipped with a variable temperature Peltier plate and a 40 mm 2° aluminum cone was used for all rheological experiments. The storage modulus (G') and loss modulus (G'') were determined as a function of temperature at an applied strain of 1.0 % and an angular frequency of 1.0 rad s^{-1}. A pre-equilibration time of 20 min at 4 °C was allowed prior to each experiment. For 10% w/w aqueous dispersions of PEG_{57}-$PHPMA_{120}$ worms, the thermal cycle was performed from 25 °C to 4 °C to 25 °C with 5 min equilibration allowed per 1 °C. For 12% w/w aqueous dispersions of PEG_{57}-$PHPMA_{65}$ worms made up in *Nutristem*, the thermal cycle was 4 °C to 37 °C to 4 °C with 2 min equilibration allowed per 1 °C.

References

1. N. J. Warren, O. O. Mykhaylyk, D. Mahmood, A. J. Ryan and S. P. Armes, *Journal of the American Chemical Society*, 2014, **136**, 1023-1033.
2. N. J. W. Penfold, A. J. Parnell, M. Molina, P. Verstraete, J. Smets and S. P. Armes, *Langmuir*, 2017, **33**, 14425-14436.
3. E. Wang, *Journal of Cellular Physiology*, 1989, **140**, 418-426.
4. J. S. Pedersen, *J. Appl. Crystallogr.*, 2000, **33**, 637-640.
5. J. S. Pedersen and P. Schurtenberger, *Macromolecules*, 1996, **29**, 7602-7612.
6. L. A. Feigin and D. I. Svergun, *Structure Analysis by Small-Angle X-Ray and Neutron Scattering*, Springer US, 1989.
7. J. Ilavsky and P. R. Jemian, *J. Appl. Crystallogr.*, 2009, **42**, 347-353.
8. Y. Takahashi and H. Tadokoro, *Macromolecules*, 1973, **6**, 672-675.
9. L. J. Fetters, D. J. Lohse, D. Richter, T. A. Witten and A. Zirkel, *Macromolecules*, 1994, **27**, 4639-4647.
